# SVM versus MAP on Accelerometer Data to Distinguish among Locomotor Activities Executed at Different Speeds

**DOI:** 10.1155/2013/343084

**Published:** 2013-11-27

**Authors:** Maurizio Schmid, Francesco Riganti-Fulginei, Ivan Bernabucci, Antonino Laudani, Daniele Bibbo, Rossana Muscillo, Alessandro Salvini, Silvia Conforto

**Affiliations:** Department of Engineering, Roma Tre University, Via Vito Volterra 62, 00146 Rome, Italy

## Abstract

Two approaches to the classification of different locomotor activities performed at various speeds are here presented and evaluated: a maximum a posteriori (MAP) Bayes' classification scheme and a Support Vector Machine (SVM) are applied on a 2D projection of 16 features extracted from accelerometer data. The locomotor activities (level walking, stair climbing, and stair descending) were recorded by an inertial sensor placed on the shank (preferred leg), performed in a natural indoor-outdoor scenario by 10 healthy young adults (age 25–35 yrs.). From each segmented activity epoch, sixteen features were chosen in the frequency and time domain. Dimension reduction was then performed through 2D Sammon's mapping. An Artificial Neural Network (ANN) was trained to mimic Sammon's mapping on the whole dataset. In the Bayes' approach, the two features were then fed to a Bayes' classifier that incorporates an update rule, while, in the SVM scheme, the ANN was considered as the kernel function of the classifier. Bayes' approach performed slightly better than SVM on both the training set (91.4% versus 90.7%) and the testing set (84.2% versus 76.0%), favoring the proposed Bayes' scheme as more suitable than the proposed SVM in distinguishing among the different monitored activities.

## 1. Introduction

With the evolution of wireless communication technology, it is now possible to use inertial sensors (Inertial Measurement Units (IMU)) to gather and transmit over the air patterns associated with different activities performed by people moving in unconstrained environments [1]. IMUs allow to collect kinematic data through miniaturized accelerometers [2], gyroscopes [3], and possibly magnetometers [4].

Restricting the analysis to accelerometers, they are popular as fall detectors [5], as means to monitor physical activity [6], and also as tools to classify among different motor activities [7, 8]. They have also been shown as good predictors of the functional capacity in healthy adults [9] and elderly people [10] and of the level of energy expenditure [11, 12]. In these specific regards, since the accuracy in the prediction strongly depends on the kind of activity [13], classification of activities is often necessary as a preliminary step for energy expenditure estimation [14].

The utility of distinguishing between activities is also apparent when, for long term monitoring, the wearable device needs to transmit data in a compact way. Following this perspective, the general communication model of having raw data to be sent continuously from the sensing devices over the air, and let the receiving unit extract relevant information from the data [15], may be a suboptimal solution. If, instead, on-board processing is available, the processing unit in each sensing unit may incorporate the function of feature extraction and subsequent activity classification [16]. In order to do this, each sensing unit will incorporate three successive functions: (1) the detection and windowing (or segmentation) of each activity epoch, (2) the extraction of the features from the windowed activity, and (3) the classification of that epoch based on a specific scheme.

As far as the classification stage is concerned, while the classification between postures is a relatively easy task [17], in the case of dynamic activities (such as different locomotion types), the classification task is more complex. This task is usually accomplished by using a multiplicity of sensors, located in different body segments and able to record the 3D components of acceleration for each segment [18]. Once an activity epoch has been detected and segmented, features from different domains are then extracted from these windowed data [19], and the classification is then performed based on a combination or a subset of these features [20]. Simple features to be extracted from windowed data include energy or amplitude parameters [21], while more complex approaches are based, for example, on dynamic programming [22], wavelet coefficients [23], and decision trees [24]. Other approaches may include particle swarm optimization, a technique that has been successfully implemented for classification and prediction in different research areas [25, 26]. Multiple accelerometers are usually added in order to improve the classification accuracy [24], even if the burdensomeness associated with the increased setup time and computational complexity makes this approach to be sought only when the increase in accuracy is significant.

In the present paper, we will thus focus on the presentation of a technique able to incorporate the functions herein described, by specifically presenting two different schemes for classification, respectively, based on the use of the maximum a posteriori approach and on a Support Vector Machine. The general objective of this work is to evaluate these two schemes in terms of their ability to distinguish among locomotor activities by using a single sensor.

The paper is structured as follows: in the second section, the structure of the two different classification schemes is presented, after giving details on the experimental procedure and providing information on the performance analysis that has been set up for evaluation. Then, we will focus on the results obtained in the experimental section, and the final section draws the conclusions.

## 2. Methods

### 2.1. Experimental Setup and Data Collection

10 healthy young adults (age 25–35 years, 4 females) volunteered in the study. They were requested to perform an 800 m path composed of different locomotor activities: walking level and incline at different slopes, stair climbing, and stair descending. They were allowed to choose their own preferred speed with which they could complete the path; in some randomly chosen sections of the path, they were requested to increase or decrease their speed, according to a command by the experimenter. In order to have the reference values, the experimenter manually noted the activity sequences.

**Figure 1:**
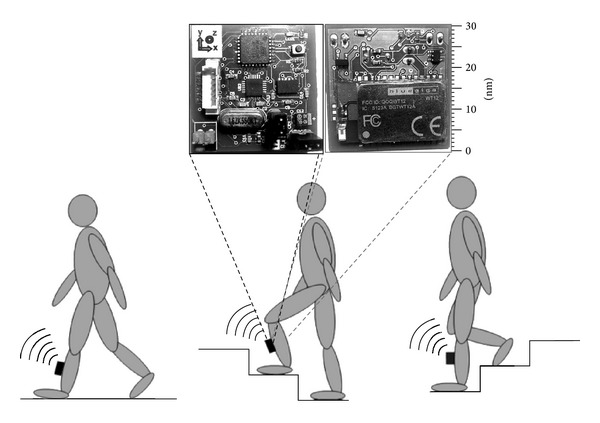
Sensor unit placement, and picture of the sensor unit: top side (left) and bottom side (right).

Data were collected through a custom-made wireless inertial sensor unit placed on the shank of the subject's preferred leg (see Figure 1); the unit is able to collect acceleration and angular rate data, as it incorporates a triaxial accelerometer (ADXL345, from Analog Devices, Inc.) and a triaxial gyroscope (ITG-3200, from Invensense, Inc.), and it includes a microcontroller (Atmega328 from Atmel Corporation) to collect and sync data from the sensors, and then send them wirelessly to a portable unit through a bluetooth transceiver (WT12, from Bluegiga Technologies Ltd.). For the purposes of this study, just the proximal-to-distal component of the accelerometer sensor was used. Data were collected at a sampling rate of 100 samples/s.

**Figure 2:**
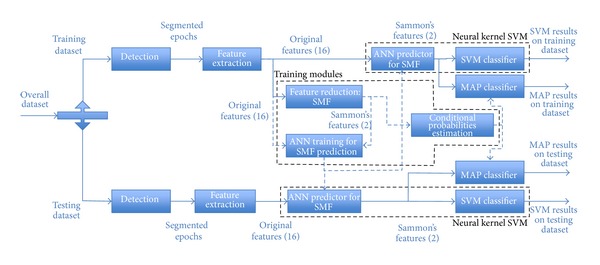
Structure of the classification schemes. Where not specifically denoted, same labels for each stage correspond to the same implementation.

The overall data processing structure will be then detailed in the following sections of the chapter.  Figure 2 shows the overall structure of the classification schemes.

### 2.2. Activity Detection and Feature Extraction

Upon digital conversion, the acceleration data were first bandpass filtered (2–20 Hz, Butterworth 4th order), underwent the segmentation process, which consisted of an integration and threshold technique [8] with first-guess threshold set at 0.35 m/s, and then were on-line adapted at 0.75 times the maximum value of the detected activity integral (100 ms window) at the previous step. Once an activity is detected, a refractory period was used (i.e., a time range when no new activities were to be detected). The first-guess refractory period was set at 600 ms, and then updated on-line at 0.5 times the duration of the last detected activity epoch. From each of the segmented activities (a walking stride or an epoch corresponding to a descending or climbing step), the procedure for the extraction of features was performed.

**Figure 3:**
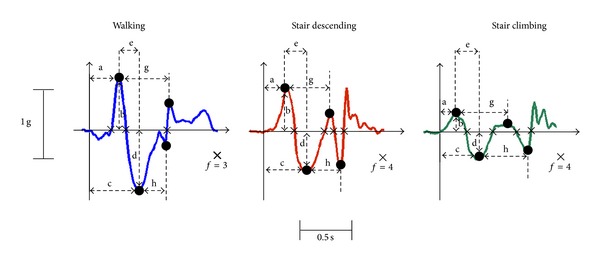
Samples of acceleration data for epochs of the three different activities performed by one participant. The corresponding features extracted from time domain ((a)–(h), please refer to text for the definition) are also shown. Four additional features extracted from the derivative in the time domain and four coming from the frequency domain are not shown here.

Sixteen different features were extracted from each detected activity: in the time domain (see Figure 3), those were: the maximum value (and its relative timing with respect to the start of the activity epoch, resp., (b) and (a) in Figure 3), the minimum value (and its relative timing, (d) and (c) resp.), the temporal distance between the maximum and the minimum value (e), the number of zero-crossings (f), the distance between two consecutive peaks (g), and the distance between two consecutive valleys (h); the maximum value of the time derivative of the epoch, and its minimum value, the maximum value of its integral (as calculated along a 100 ms window), and its minimum value; in the frequency domain, for each activity epoch, the temporal variation of its mean frequency was calculated, according to [27], and its minimum and maximum values, both in linear and logarithmic scale.

These 16 features were chosen in this way, as they were able to represent data variability on a different population sample performing similar activities [28].

### 2.3. Feature Reduction and Training Data Use

**Figure 4:**
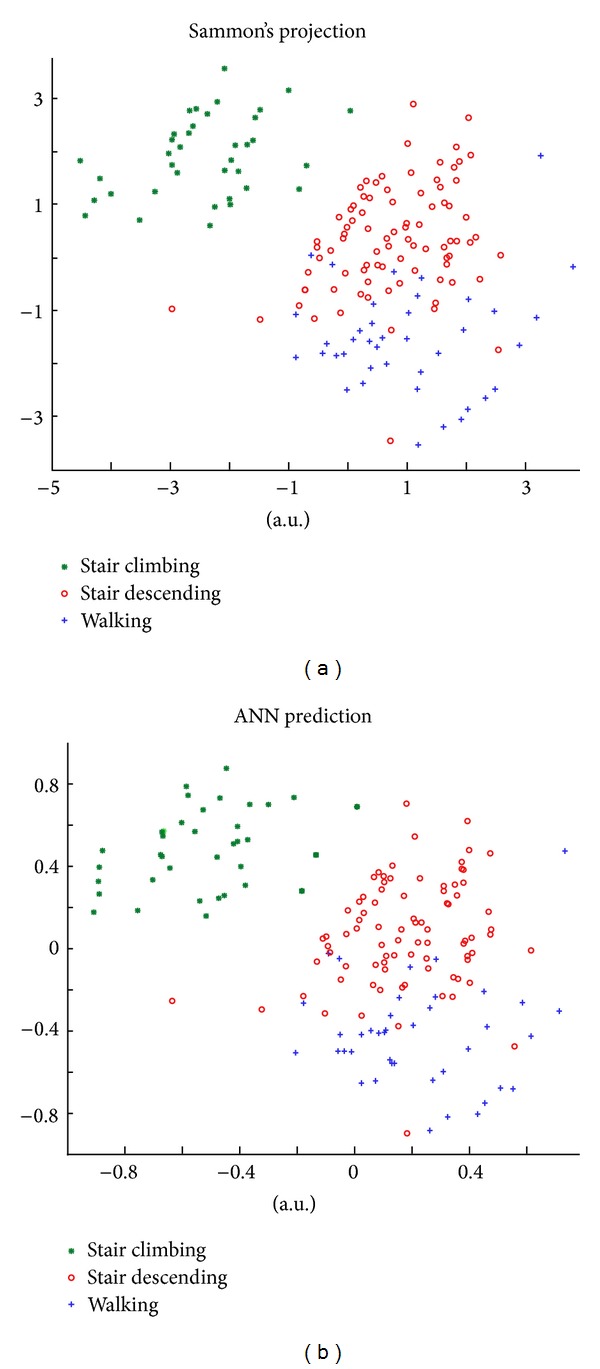
Excerpt of Sammon's features, as output from the SMF (left panel), and as estimated from the ANN (right panel), across three locomotor activities, coded by color and marker. Differences in scale were not detrimental to the successive processing phases.

In order to reduce the number of features (yet maintaining relevant information), Sammon's Mapping Function (SMF, [29]) was applied to the 16-dimensional feature set, that was mapped into a 2D output space. Nonlinear mapping was preferred to other linear factorization methods, as it qualitatively showed better results than PCA on a subsample of the training dataset. Since the mapping procedure is a recursive one, and the input-output relation cannot be determined analytically, an Artificial Neural Network (Multilayer Perceptron, one hidden layer with 40 neurons) was trained to mimic its nonlinear behavior. ANNs are one of the possible choices to solve MIMO problems that cannot be determined analytically [30, 31]. Out of the overall dataset that was used, 15% of its feature data points were used for the training of the ANN able to mimic the SMF behavior, with the same procedure that was used in [28] and in [32]. This 15% was randomly extracted from epochs of all the subjects, in order to maximize the generalization ability of the system. The ANN was trained through Levenberg-Marquardt [33] backpropagation, following the same procedure proposed in [34], and the ANN was deemed as trained if the Mean Square Error fell below 0.1%. This actually happened with approximately 10000 iterations.  Figure 4 shows the results of the mapping estimation through the ANN: as expected, ANN was able to accurately predict Sammon's features in the training dataset, thanks to its ability to adapt to different mapping and approximation [35] problems, as shown, for example, in [36, 37]. Cross correlation of the training set data, between features coming from SMF and the ones estimated through the ANN, resulted to be higher than 0.98.

### 2.4. Classifiers

Once the two features were estimated with the ANN, the following stage consisted of classifying among the different locomotor activities. In order to complete this, two different classifiers were implemented: the first relies on the representation of Bayes' Theorem and estimates the activity based on a maximum a posteriori (MAP) criterion, and it will be called as MAP in the following; the second makes use of the Support Vector Machines, and it will be denoted as SVM in the following. The structure of both the classifiers is detailed in the following subsections.

#### 2.4.1. Maximum A Posteriori (MAP) Approach

According to Bayes' theorem, we will determine the estimated activity $\hat{\text{act}}$, based on the calculation of the conditional probabilities associated with the different locomotor activities act_*i*_, and the current value of Sammon's feature vector (2D) *s*, according to the following equations:

(1) $$\begin{array}{c} P\left(\text{ac}{\text{t}}_{i}\;|\;s\right)=p\left(s\;|\;\text{ac}{\text{t}}_{i}\right)*\frac{P\left(\text{ac}{\text{t}}_{i}\right)}{p\left(s\right)},\\ \hat{\text{act}}=\arg\;\underset{i\in I}{\max}P\left(\text{ac}{\text{t}}_{i}\;|\;s\right), \end{array}$$

where *I* represents the domain of possible activities to be classified.

In order for the MAP criterion to be utilized, we thus need the conditional probabilities *p*(*s* | act_*i*_) and the prior probabilities *P*(act_*i*_). The first ones were hypothesized as coming from a 2D Gaussian probability density function, with first- and second-order moments equal to the values obtained from the training dataset. The prior probabilities were hypothesized as equally distributed. In the current case, this choice slightly underestimated the priors for walking activities in the used set, but we chose this criterion, in order for the classifier to be more general in classification capabilities. The MAP classifier also incorporated an update rule for the prior probabilities to be used in the current step, which was based on replacing, within a sample vector of 240 activity identifiers, the oldest sample for the classified activity with the one classified at the previous step.

#### 2.4.2. Support Vector Machine

In the case of the Support Vector Machine, there was no kernel use, even if the transformation from the 16-dimensional space of the original features into the 2D predicted Sammon's features may be considered as a kernel *perse*, as it incorporated a nonlinear mapping to be considered as a kernel trick, with the major difference that, in this case, the new space is low-dimensional as compared to the original one.

With regard to the implementation, since we chose a low-dimensional space for the SVM to be used, linear classification was suboptimal, and we used a penalty coefficient to take into account misclassifications; concerning the optimization, we used the Mitchell-Demyanov-Malozemov (MDM) algorithm [38], with a regularization constant value of 5. Given that three classes were to be used, multiclass condition was solved by using the one versus one conditions, with max-wins voting criterion.

### 2.5. Performance Indicators

In order to evaluate the performance of both the classifying schemes, we calculated the classification rate for both the training set and the testing set. Confusion matrix and normalized mutual information [39] were also reported for the testing set. With ten subjects performing the requested walk path, a total of approximately 12000 activity epochs were collected.  Table 1 shows the overall number of activities as split among the different kinds and speeds. It is here to be highlighted that speed was considered as a confounding factor and not as a variable on which classification was made. This is to mimic a natural scenario, where differences in energy associated with each epoch can be extracted directly on the data of each epoch, once the classification is made.

## 3. Results

Classification rates for the training set and the testing set are reported in Table 2. As expected, both classifiers perform quite accurately in the training set, while there is a marked difference between MAP and SVM in the case of the testing set that favored the first as compared to the second.

Performance in the training set is almost independent from the activity kind. Moreover, as reported in Table 3, misclassification in the testing set more frequently occurs between walking strides and strides of descending stairs.

## 4. Discussion and Conclusions

Classification rates for both schemes were, on average, good on the training dataset. Misclassifications, which occurred most frequently with walking and stair descending, may be associated with the fact that the features extracted from these two activities are on average more similar than the ones coming from stair climbing (see Figure 3); this similarity may be even more exacerbated in the transition activities (initiating a stair climbing or descending after walking or vice versa).

For the testing dataset, the maximum a posteriori approach performed better than the SVM. We speculate that, based on the results obtained in the training dataset, the structure of the MAP approach implemented in this paper has a higher generalization ability than the SVM in classifying these activities, since it includes an adaptation that updates the prior probabilities based on the history of the classification. This has not been implemented in the SVM approach, which may consequently have a decreased ability to track differences in the extracted features as a consequence of subjective and environmental factors (fatigue and variations in speed).

As far as the overall performance is concerned, classification rates are similar to those reported in [40] and in [18], where different accelerometer configurations and features were tested, with classification accuracies lying in the range 68%–97% for triaxial sensors. It is here to be highlighted that the obtained classification rates have been based on the use of just a single component of an accelerometer. This was done in order to check whether on-board processing might be considered as a viable alternative to continuous raw data communication. It is predicted that, if multiple instances of the same classification schemes may be adopted on multiple sensors placed on different body segments, the portable unit may produce better results, possibly based on a max-wins voting criterion.

As for the current implementation, the structure is relatively easy to be implemented on-board; the detection and feature extraction section is relatively light in terms of computational complexity (with only frequency features slightly weighing in), and, once the training modules are appropriately determined based on an adequate number of subjects, running the classifying modules and determining the decisions is a pretty straightforward step for both approaches: for MAP, it corresponds to running the ANN predictor and calculating the posterior probabilities and for SVM, it corresponds to running the ANN predictor and then applying the hyperplane (in the current case of 2D representation, a line) estimated through the SVM on the training dataset.

In the future it would be useful to insert some update rules in the SVM classification scheme, as it has been done in the MAP approach, to let it take into account the temporal variations of the accelerometer patterns in a long-term scenario.

In conclusion, the availability of different classification schemes that can be profitably applied to single sensor data may help designing body sensor networks where the classification may be done on-board in each node, so that the data throughput can be substantially reduced, and the possibility to have accurate parameters for long-term monitoring can be pursued.

## Figures and Tables

**Table 1:** Activity epochs for the dataset.

Activity	Requested slow speed	Self-selected speed	Requested fast speed	Total
Walking	720	4265	463	5448
Stair climbing	322	2742	340	3404
Stair descending	296	2678	326	3300

Total	1338	9685	1129	12152

**Table 2:** Classification results.

Training set	SVM	MAP
Walking (%)	90.4	91.8
Stair climbing (%)	91.3	90.9
Stair descending (%)	90.1	90.0

Testing set	SVM	MAP

Walking (%)	77.2	85.1
Stair climbing (%)	77.7	87.2
Stair descending (%)	74.2	81.3

**Table 3:** Confusion matrix and Normalized Mutual Information (NMI) for the testing set.

	Predicted SVM			(NMI = 0.3550)
	Activity	Walking	Stair climbing	Stair descending
Actual	Walking (%)	77.2	8.1	14.6
	Stair climbing (%)	10.2	77.7	12.1
	Stair descending (%)	15.8	9.9	74.2

	Predicted MAP			(NMI = 0.5134)
	Activity	Walking	Stair climbing	Stair descending

Actual	Walking (%)	85.1	4.8	10.1
	Stair climbing (%)	6.6	87.2	6.3
	Stair descending (%)	12.8	5.8	81.3

## References

[1] Patel S, Park H, Bonato P, Chan L, Rodgers M (2012). A review of wearable sensors and systems with application in rehabilitation. *Journal of NeuroEngineering and Rehabilitation*.

[2] Kavanagh JJ, Menz HB (2008). Accelerometry: a technique for quantifying movement patterns during walking. *Gait and Posture*.

[3] Tong K, Granat MH (1999). A practical gait analysis system using gyroscopes. *Medical Engineering and Physics*.

[4] O’Donovan KJ, Kamnik R, O’Keeffe DT, Lyons GM (2007). An inertial and magnetic sensor based technique for joint angle measurement. *Journal of Biomechanics*.

[5] Bourke AK, O’Brien JV, Lyons GM (2007). Evaluation of a threshold-based tri-axial accelerometer fall detection algorithm. *Gait and Posture*.

[6] Troiano RP, Berrigan D, Dodd KW, Mâsse LC, Tilert T, Mcdowell M (2008). Physical activity in the United States measured by accelerometer. *Medicine and Science in Sports and Exercise*.

[7] Karantonis DM, Narayanan MR, Mathie M, Lovell NH, Celler BG (2006). Implementation of a real-time human movement classifier using a triaxial accelerometer for ambulatory monitoring. *IEEE Transactions on Information Technology in Biomedicine*.

[8] Muscillo R, Conforto S, Schmid M, Caselli P, D’Alessio T Classification of motor activities through derivative dynamic time warping applied on accelerometer data.

[9] Bouten CVC, Verboeket-van de Venne WPHG, Westerterp KR, Verduin M, Janssen JD (1996). Daily physical activity assessment: comparison between movement registration and doubly labeled water. *Journal of Applied Physiology*.

[10] Meijer EP, Goris AHC, Wouters L, Westerterp KR (2001). Physical inactivity as a determinant of the physical activity level in the elderly. *International Journal of Obesity*.

[11] Yoshioka M, Ayabe M, Yahiro T (2005). Long-period accelerometer monitoring shows the role of physical activity in overweight and obesity. *International Journal of Obesity*.

[12] Bouten CVC, Westerterp KR, Verduin M, Janssen JD (1994). Assessment of energy expenditure for physical activity using a triaxial accelerometer. *Medicine and Science in Sports and Exercise*.

[13] Chen KY, Sun M (1997). Improving energy expenditure estimation by using a triaxial accelerometer. *Journal of Applied Physiology*.

[14] Staudenmayer J, Pober D, Crouter S, Bassett D, Freedson P (2009). An artificial neural network to estimate physical activity energy expenditure and identify physical activity type from an accelerometer. *Journal of Applied Physiology*.

[15] Hao Y, Foster R (2008). Wireless body sensor networks for health-monitoring applications. *Physiological Measurement*.

[16] Jovanov E, Milenkovic A, Otto C, De Groen PC (2005). A wireless body area network of intelligent motion sensors for computer assisted physical rehabilitation. *Journal of NeuroEngineering and Rehabilitation*.

[17] Hendelman D, Miller K, Baggett C, Debold E, Freedson P (2000). Validity of accelerometry for the assessment of moderate intensity physical activity in the field. *Medicine and Science in Sports and Exercise*.

[18] Preece SJ, Goulermas JY, Kenney LPJ, Howard D (2009). A comparison of feature extraction methods for the classification of dynamic activities from accelerometer data. *IEEE Transactions on Biomedical Engineering*.

[19] Preece SJ, Goulermas JY, Kenney LPJ, Howard D, Meijer K, Crompton R (2009). Activity identification using body-mounted sensors—a review of classification techniques. *Physiological Measurement*.

[20] Pirttikangas S, Fujinami K, Nakajima T Feature selection and activity recognition from wearable sensors.

[21] Veltink PH, Bussmann HBJ, De Vries W, Martens WLJ, Van Lummel RC (1996). Detection of static and dynamic activities using uniaxial accelerometers. *IEEE Transactions on Rehabilitation Engineering*.

[22] Muscillo R, Schmid M, Conforto S, D'Alessio T (2011). Early recognition of upper limb motor tasks through accelerometers: real-time implementation of a DTW-based algorithm. *Computers in Biology and Medicine*.

[23] Najafi B, Aminian K, Paraschiv-Ionescu A, Loew F, Büla CJ, Robert P (2003). Ambulatory system for human motion analysis using a kinematic sensor: monitoring of daily physical activity in the elderly. *IEEE Transactions on Biomedical Engineering*.

[24] Pärkkä J, Ermes M, Korpipää P, Mäntyjärvi J, Peltola J, Korhonen I (2006). Activity classification using realistic data from wearable sensors. *IEEE Transactions on Information Technology in Biomedicine*.

[25] Wu D, Warwick K, Ma Z (2010). Prediction of parkinson’s disease tremor onset using a radial basis function neural network based on particle swarm optimization. *International Journal of Neural Systems*.

[26] Riganti Fulginei F, Salvini A (2009). Hysteresis model identification by the flock-of-starlings optimization. *International Journal of Applied Electromagnetics and Mechanics*.

[27] Conforto S, D’Alessio T (1999). Real time monitoring of muscular fatigue from dynamic surface myoelectric signals using a complex covariance approach. *Medical Engineering and Physics*.

[28] Muscillo R, Schmid M, Conforto S, D’Alessio T (2010). An adaptive Kalman-based Bayes estimation technique to classify locomotor activities in young and elderly adults through accelerometers. *Medical Engineering and Physics*.

[29] Sammon JW (1969). A non-linear mapping for data structure analysis. *IEEE Transactions on Computers*.

[30] Fulginei FR, Laudani A, Salvini A, Parodi M (2013). Automatic and parallel optimized learning for neural networks performing mimo applications. *Advances in Electrical and Computer Engineering*.

[31] Fulginei FR, Salvini A, Parodi M (2012). Learning optimization of neural networks used for MIMO applications based on multivariate functions decomposition. *Inverse Problems in Science and Engineering*.

[32] Fulginei FR, Salvini A (2012). Neural network approach for modelling hysteretic magnetic materials under distorted excitations. *IEEE Transactions on Magnetics*.

[33] Marquardt D (1963). An algorithm for least-squares estimation of nonlinear parameters. *SIAM Journal on Applied Mathematics*.

[34] Gneo M, Schmid M, Conforto S, D'Alessio T (2012). A free geometry model-independent neural eye-gaze tracking system. *Journal of Neuroengineering and Rehabilitation*.

[35] Capizzi G, Coco S, Giuffrida C, Laudani A (2004). A neural network approach for the differentiation of numerical solutions of 3-D electromagnetic problems. *IEEE Transactions on Magnetics*.

[36] Del Vecchio P, Salvini A (2000). Neural Network and Fourier Descriptor macromodeling dynamic hysteresis. *IEEE Transactions on Magnetics*.

[37] Salvini A, Coltelli C (2001). Prediction of dynamic hysteresis under highly distorted exciting fields by neural networks and actual frequency transplantation. *IEEE Transactions on Magnetics*.

[38] Mitchell BF, Dem’yanov VF, Malozemov VN (1974). Finding the point of a polyhedron closest to the origin. *SIAM Journal on Control*.

[39] Cai D, He X, Han J (2005). Document clustering using locality preserving indexing. *IEEE Transactions on Knowledge and Data Engineering*.

[40] Chan H, Yang M, Wang H (2013). Assessing gait patterns of healthy adults climbing stairs employing machine learning techniques. *International Journal of Intelligent Systems*.

